# Nitrogen use efficiency in crops under salt stress: from molecular networks to intelligent breeding

**DOI:** 10.3389/fpls.2026.1922452

**Published:** 2026-08-10

**Authors:** Ranran Liu, Longyu Wang, Shulei Wang, Yanting Wang, Like Liu

**Affiliations:** College of Agriculture and Biology, Liaocheng University, Liaocheng, China

**Keywords:** carbon–nitrogen metabolism, genome editing, intelligent breeding, nitrogen use efficiency, NRT/NPF transporters, salt stress

## Abstract

Soil salinization threatens global arable land and agricultural sustainability, severely reducing crop nitrogen use efficiency (NUE) by disrupting root ammonium and nitrate fluxes, impairing nitrogen-assimilation enzymes, and disrupting carbon–nitrogen (C–N) balance. This review synthesizes recent advances in the coordination of salt-stress signaling and nitrogen homeostasis in plants. Two mechanistically distinct regulatory axes have recently been proposed. In one, a nitrate transporter acts as a dual sensor for nitrate and abscisic acid (ABA); in the other, SOS kinase-mediated phosphorylation of an ammonium transporter maintains ammonium uptake under Na^+^ stress. In addition, rapid post-translational regulatory mechanisms, including reversible protein phosphorylation and *S*-nitrosylation of nitrate reductase, can fine-tune nitrogen fluxes shortly after salt exposure. These findings inform a four-tier closed-loop conceptual framework comprising signal perception, transport reprogramming, metabolic redistribution, and genetic redesign. The framework yields three testable predictions: the sequential activation of regulatory tiers; a quantitative relationship between Ca^2+^ signal amplitude and the extent of C–N metabolic redistribution; and salt-concentration thresholds that distinguish basal homeostatic buffering from full adaptive reprogramming. Translation of this framework to field crops requires an integrated breeding pipeline that combines multi-environment quantitative trait locus (QTL) mapping, pan-genome-enabled genome-wide association studies, genomic selection for minor-effect alleles, and multiplex CRISPR editing coupled with stress-inducible synthetic promoters to pyramid favorable traits while minimizing yield penalties. A major unresolved challenge is to resolve the dynamic protein–metabolite networks that govern growth–defense trade-offs under combined salinity and nitrogen limitation. The integration of single-cell transcriptomics, isotope-based metabolic flux analysis, and machine-learning-assisted phenomics may help link genotypic variation to agronomic performance in salinized agroecosystems.

## 1 Introduction

Soil salinization is a major abiotic constraint on global agricultural sustainability. Salinity impairs nitrogen use efficiency through ion toxicity, osmotic stress, and oxidative damage (Munns and Tester, 2008). Over 20% of global arable land affected, yet the standard agronomic countermeasure (excessive nitrogen fertilization) does not restore yields proportionally. Instead, it triggers a deleterious feedback loop: low NUE under salt stress leads to nitrogen over‑application, which further aggravates secondary salinization and ecological eutrophication (Gong et al., 2020). Breaking this cycle requires understanding the molecular crosstalk between salt signaling and nitrogen metabolism to engineer cultivars that combine stress resilience with high NUE. This is therefore strategically important for food security and sustainable agriculture.

The regulatory intersection of salt and nitrogen operates through both physical competition and reciprocal signaling. High external Na^+^ and Cl^–^ competitively inhibit root transporters for NH_4_^+^ and NO_3_^–^ (Li et al., 2018; Hessini et al., 2019), while salt-induced osmotic stress reduces root hydraulic conductivity and restricts nitrogen translocation to shoots (Hessini et al., 2019). Conversely, nitrogen availability dynamically calibrates salt tolerance. Nitrate-dependent nitric oxide (NO) production via nitrate reductase (NR) primes antioxidant defenses, while optimized NH_4_^+^ supply maintains K^+^/Na^+^ homeostasis through the Salt Overly Sensitive (SOS) pathway (Meng et al., 2016). This reciprocal modulation defines the physiological boundaries within which crops navigate the growth–defense trade-off.

Recent mechanistic advances have improved our understanding of this crosstalk. Notably, recent studies have reported specific kinase-transporter physical interactions that may coordinate ion homeostasis and nitrogen uptake, such as the SOS2-mediated phosphorylation of AMT1;1 under Na^+^ stress (Ma et al., 2025a). Concurrently, the integration of stress and nutrient signaling has been proposed to occur at the plasma membrane, with evidence suggesting that OsNRT1.1B functions as a dual receptor for nitrate and ABA (Ma et al., 2025b). These findings, while mechanistically intriguing, require independent validation across species. Other studies have highlighted the reactive oxygen species (ROS)–Ca^2+^–mitogen-activated protein kinase (MAPK) cascade and NO-mediated protein *S*‑nitrosylation as core transducers linking membrane perception to metabolic rewiring (Mangal et al., 2023; Wei et al., 2024a).

Despite these advances, three major gaps remain. First, the field lacks a unified mechanistic model; the current literature remains fragmented and focuses on individual transporters rather than a continuous regulatory network. Second, the systematic role of post-translational modifications (PTMs), specifically how phosphorylation and *S*‑nitrosylation act as dynamic rheostats of rate-limiting enzymes like NR and glutamine synthetase (GS), remains unresolved. Third, a substantial translational gap exists between molecular breeding and field performance. Because most genetic studies are conducted under controlled conditions, markers identified in the laboratory often fail to perform consistently in heterogeneous saline fields due to genotype × environment (G × E) interactions (Xing et al., 2023; Budhlakoti et al., 2022).

**Figure 1:**
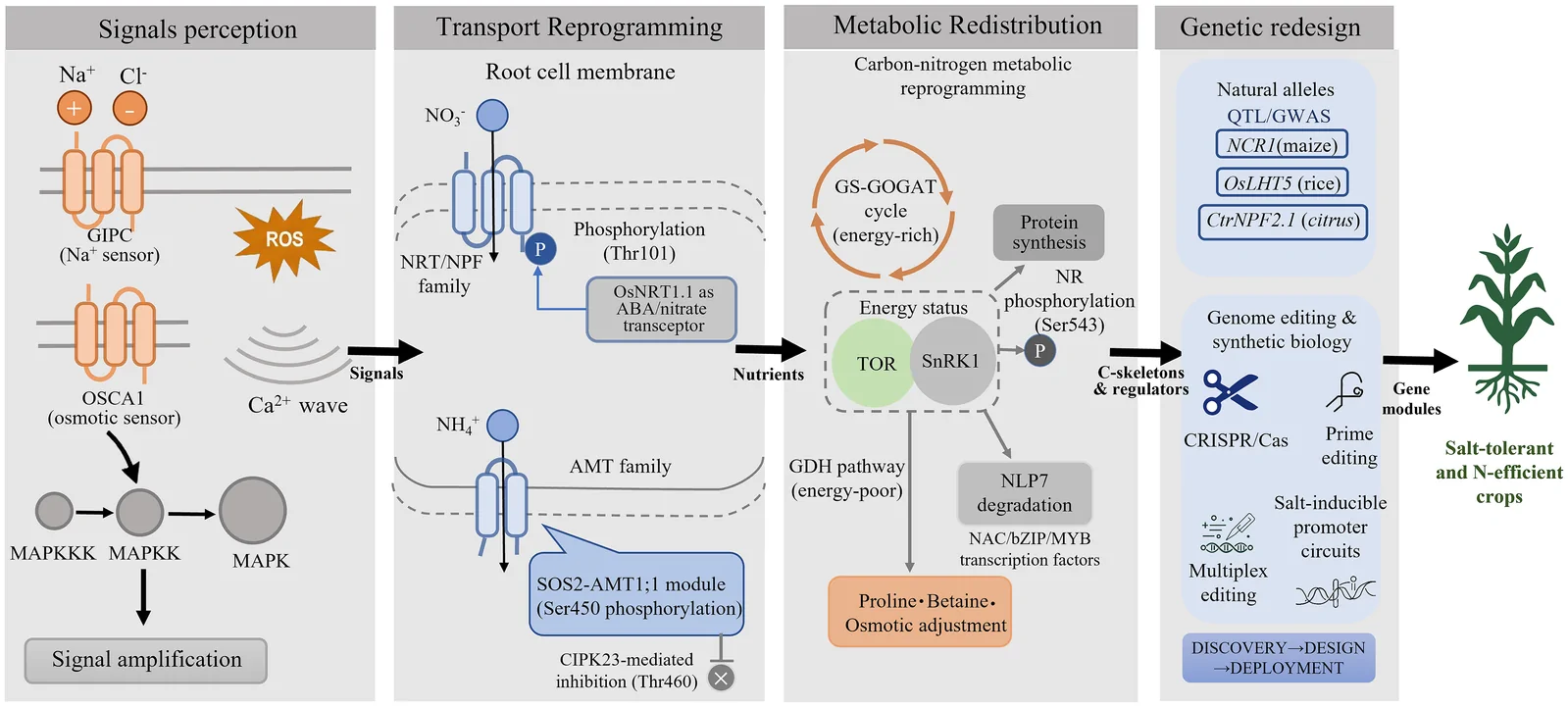
Conceptual four-tier closed-loop framework for salt-nitrogen crosstalk, featuring bidirectional feedback rather than linear signal cascades. The model comprises four functionally coupled phases. (1) Signal perception: Plasma membrane sensors (e.g., GIPC, OSCA1) detect ionic and osmotic cues, amplifying them via ROS–Ca^2+^–MAPK cascades. (2) Transport reprogramming: Root NRT/NPF and AMT transporters mediate nitrogen acquisition, fine‑tuned by phosphorylation switches (e.g., NRT1.1 at Thr101) and kinase‑transporter coupling (e.g., SOS2–AMT1;1). OsNRT1.1B functions as a dual nitrate/ABA transceptor, directly linking nutrient status to stress signaling. (3) Metabolic redistribution: The TOR–SnRK1 energy hub maintains the balance between the primary GS‑GOGAT cycle and the ATP‑independent GDH shunt, driving the reallocation of nitrogen from growth to osmoprotectant biosynthesis (proline, betaine). NR phosphorylation at Ser‑543 acts as a rapid rheostat on this process. (4) Genetic redesign: Elite natural alleles (e.g., *NCR1*, *OsLHT5*, *CtrNPF2.1*) are identified via QTL/GWAS and deployed through multiplex genome editing and synthetic biology circuits, following a DISCOVERY→DESIGN→DEPLOYMENT pipeline to create salt‑tolerant, nitrogen‑efficient crops. Partial visual components in this figure are adapted from BioGDP (https://biogdp.com) (Jiang et al., 2025).

To resolve these bottlenecks, we synthesize current evidence to propose a closed‑loop biological model alongside a translational breeding strategy. The biological model comprises four sequential regulatory phases: (1) Signal Perception, in which membrane receptors (e.g., OsNRT1.1B) and ion channels transduce initial salt/nitrate cues; (2) Transport Reprogramming, featuring transporter–kinase modules (e.g., SOS2–AMT1;1) that secure nitrogen influx under ionic stress; (3) Metabolic Redistribution, in which carbon–nitrogen flux is rewired via energy-sensing hubs like TOR–SnRK1 (Target of Rapamycin–SNF1-related protein kinase 1); and (4) Transcriptional Feedback, which closes the loop by adjusting cellular homeostasis. The hierarchical architecture and core regulatory nodes of this framework are summarized in **Figure 1**. This four-tier structure is a conceptual organizing framework rather than a fully quantified mechanistic model. The loop architecture differs from linear signaling chains through two-way regulatory feedback: late-stage transcriptional outputs feed back to tune upstream plasma transporters, while cellular metabolic signals modulate early stress sensors. This framework generates three testable distinctions from prior linear models: (1) stress response follows a fixed sequence of tier activation; (2) calcium signal magnitude correlates with downstream metabolic shift strength; (3) salt concentration thresholds separate mild homeostatic buffering from full adaptive reprogramming. Comparable quantitative data on fluxes and thresholds across crops are not yet available.

Among these regulatory phases, the OsNRT1.1B dual-sensing and SOS2–AMT1;1 phosphorylation modules derive from very recent single-laboratory reports and, as noted above, await independent validation. More broadly, the four-tier closed-loop mechanistic framework proposed in this review is primarily constructed from molecular evidence generated in rice (*Oryza sativa*) and *Arabidopsis thaliana*. This taxonomic imbalance reflects the current landscape of published research: complete biochemical validations of kinase-transporter phosphorylation, dual nutrient-stress sensing and dynamic PTM cascades are overwhelmingly restricted to these two model systems. For staple crops (wheat and maize) and horticultural species (soybean and tomato), existing datasets are mostly limited to transcriptome profiling and whole-plant physiological phenotyping, with few *in vitro* and *in vivo* functional characterizations of the core regulatory modules summarized in Sections 3–5. Despite these limitations, the hierarchical signaling logic (ROS–Ca^2+^ signal amplification, phosphorylation-based transporter regulation, and TOR–SnRK1 energy balancing) is evolutionarily conserved across angiosperms, providing a broad conceptual scaffold. However, lineage‑specific functional divergence exists among nitrogen transporter paralogs, stress response thresholds and tissue‑specific metabolic rewiring, direct extrapolation of rice/*Arabidopsis*-derived regulatory mechanisms cannot guarantee equivalent phenotypic outputs without independent crop‑specific validation.

Building upon this mechanistic loop, this review synthesizes how targeted PTMs act as dynamic regulators that rapidly tune this network. Ultimately, we outline a translational roadmap linking high-resolution QTL mapping, single-cell multi-omics, machine learning, and multiplex CRISPR editing to transition from piecemeal discovery to mechanism-based development of climate-resilient, nitrogen-efficient crops.

## 2 Physiological and ecological basis of salt stress–nitrogen nutrition crosstalk

**Figure 2:**
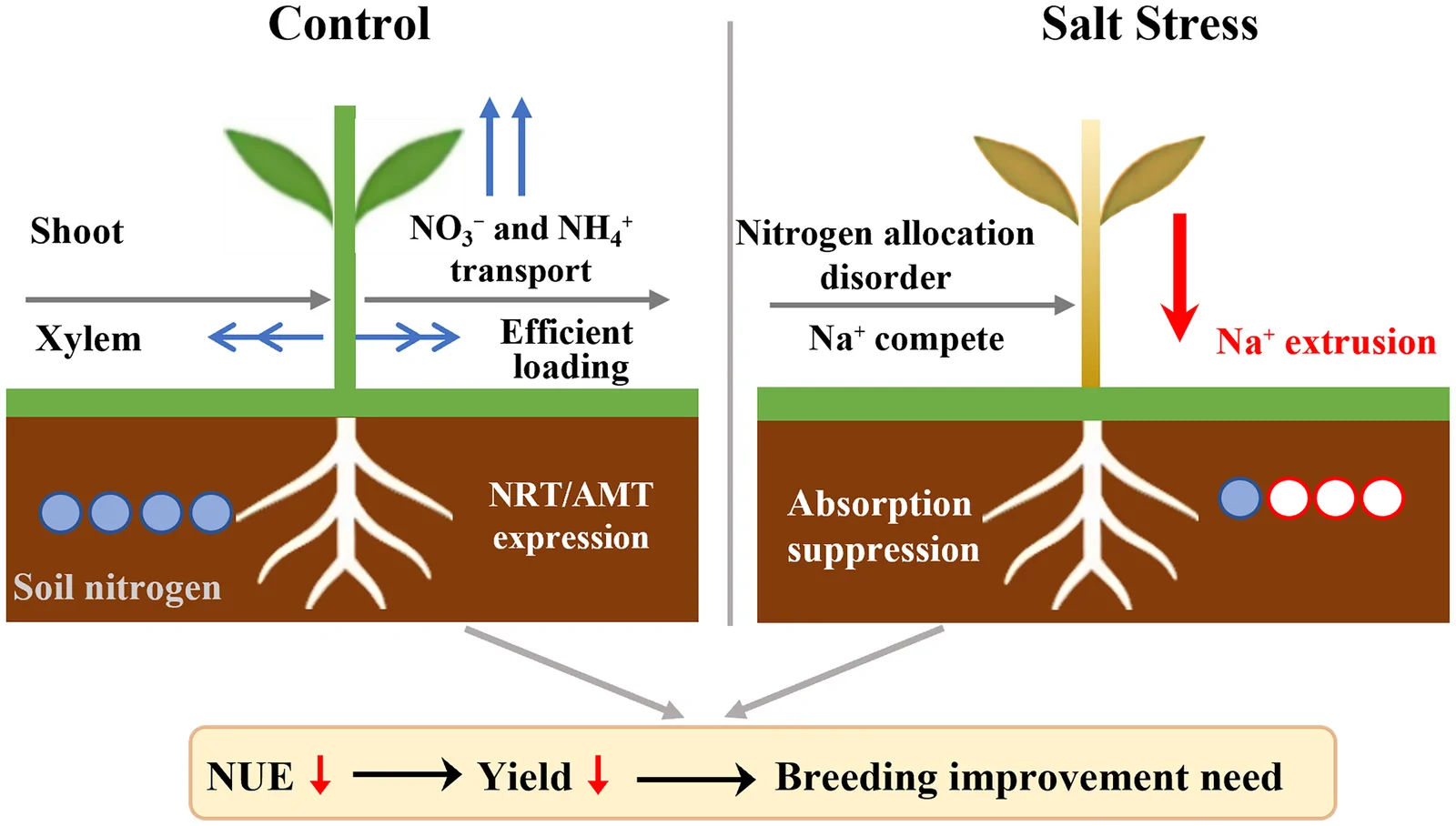
Graphical overview of nitrogen uptake, transport, and allocation in crops under normal versus salt stress conditions. Left panel (Normal conditions): Roots actively take up NO_3_^–^ and NH_4_^+^ via NRT/AMT transporters, followed by efficient xylem loading and long‑distance transport to the shoot, where nitrogen is allocated to support vegetative growth. Right panel (Salt stress): High external Na^+^ competitively inhibits root transporter activity, suppresses net nitrogen uptake, impairs xylem loading, and triggers a systemic reallocation of nitrogen away from growth toward defense (e.g., Na^+^ extrusion). Bottom panel: The cascade from salt‑impaired nitrogen uptake to reduced NUE and subsequent yield penalties establishes the agronomic imperative for breeding improvement. Blue arrows indicate normal N metabolic flux; red arrows indicate processes disrupted by salinity.

The physiological interaction between salt stress and nitrogen nutrition establishes the phenotypic baseline for the closed-loop biological model proposed in this review. High salinity impairs root nitrogen acquisition and whole-plant allocation through coupled ionic toxicity and osmotic stress. The cascade from salt-impaired nitrogen uptake to reduced NUE and yield penalties, culminating in the need for breeding improvement, is illustrated in **Figure 2**, providing a macroscopic foundation for subsequent mechanistic dissection.

### 2.1 Inhibitor effects of salt stress on nitrogen utilization and differential regulation of nitrogen forms

Salt stress diminishes crop NUE via three compounding physiological constraints: (1) Ionic antagonism: High external Na^+^ competitively antagonizes NH_4_^+^ uptake at the root plasma membrane via monovalent cation transporters, while excessive Cl^–^ outcompetes NO_3_^–^ for anion channels, synergistically restricting net inorganic nitrogen influx. (2) Root architecture remodeling: Salinity suppresses primary root elongation and lateral root initiation, diminishing the spatial foraging capacity for heterogeneous soil nitrogen pools. (3) Metabolic inhibition: Hyperaccumulated cytosolic ions directly disrupt the structural integrity and catalytic efficiency of rate-limiting nitrogen assimilatory enzymes, notably NR and GS (Nazir et al., 2023; Phan et al., 2023a). Consequently, both absorptive NUE (aNUE) and agronomic NUE (agNUE) decline in salinized agroecosystems.

The metabolic consequences of salinity are linked to the predominant inorganic nitrogen form (NO_3_^–^ vs. NH_4_^+^). In staple crops such as rice, NO_3_^–^ nutrition typically mitigates salt stress via dual mechanisms: NO_3_^–^ influx competitively restricts Cl^–^ accumulation; NO_3_^–^ acts as a signaling molecule to induce NR-dependent NO production, which subsequently upregulates the K^+^ transporter OsAKT1 to maintain a favorable cytosolic K^+^/Na^+^ ratio (Teng et al., 2025). Conversely, NH_4_^+^ and Cl^–^ often exhibit synergistic root-to-shoot translocation, accelerating Cl^–^ hyperaccumulation and promotes leaf senescence (Liu et al., 2020). However, this paradigm is highly species-dependent. For instance, maize exhibits superior osmotic adjustment under sole NH_4_^+^ nutrition compared with NO_3_^–^, underscoring that the optimal nitrogen formulation varies with genetic background and stress severity (Hessini et al., 2019; Noor et al., 2024).

Crucially, adaptive evolution has equipped specific nitrogen transporters with dual functions in nutrient flux and ion homeostasis, positioning them as central hubs in salt-nitrogen crosstalk. At the plasma membrane, the rice transceptor OsNRT1.1B integrates nitrate availability directly with ABA-mediated stress signaling (Ma et al., 2025b), while the SOS2 kinase selectively phosphorylates AMT1;1 to sustain ammonium uptake under Na^+^-induced depolarization (Ma et al., 2025a). Intracellularly, the regulatory module also operates at the tonoplast. In trifoliate orange (*Citrus trifoliata*), the CtrNAC019–CtrNPF2.1 module actively drives vacuolar Cl^–^ sequestration while facilitating NO_3_^–^ efflux into the cytosol, decoupling ion detoxification from nitrogen assimilation (Zhao et al., 2026). Overlooking these physiological mechanisms in agricultural practice, such as attempting to overcome salt-induced yield penalties through indiscriminate nitrogen over-application, not only fails to rescue productivity but also triggers severe nitrate leaching and environmental eutrophication (Ribeiro et al., 2020).

### 2.2 Carbon‑nitrogen metabolic reprogramming and the physiological cost of the growth-defense trade‑off

The constraints on nitrogen uptake imposed by salt stress do not curtail nutrient availability; they trigger a systemic reprogramming of C–N metabolism. This reprogramming reflects a strategic reallocation of nitrogen from growth-oriented anabolism (e.g., photosynthetic protein synthesis and ribosomal biogenesis) toward defense-oriented metabolism (e.g., osmoprotectants and ROS scavengers). This metabolic shift constitutes the biochemical hallmark of the plant growth-defense trade-off under salinity.

Insufficient carbon skeleton supply acts as the primary bottleneck driving this C–N metabolic rewiring. Under high salinity, disruption of chloroplast ultrastructure and suppression of photosystem II (PSII) and Rubisco activities reduce CO_2_ fixation efficiency, whereas moderate salinity can enhance photosynthetic performance in certain halophytic species (Li et al., 2025a). This photosynthetic decline under severe salinity directly limits the availability of 2-oxoglutarate (2-OG), the obligate carbon acceptor for the glutamine synthetase/glutamate synthase (GS/GOGAT) nitrogen assimilation cycle, consequently curtailing the primary nitrogen assimilation flux (Vergara-Diaz et al., 2024). Confronted with this energy and carbon shortfall, plants often upregulate low-affinity, ATP-independent alternative pathways, notably the glutamate dehydrogenase (GDH) shunt, to sustain basal nitrogen metabolism. However, this compensatory mechanism exacts a cost: the inherently low catalytic efficiency of the GDH pathway further depresses whole-plant NUE (Wang et al., 2012).

This carbon deficit triggers a feedback loop in nitrogen allocation. Significant nitrogen pools are diverted into the biosynthesis of amino acid-derived osmoprotectants, particularly glutamate-derived proline, exacerbating the nitrogen deficiency required for vegetative development. In turn, systemic nitrogen insufficiency restricts the synthesis of chlorophyll and light-harvesting complex proteins, leading to a further decline in carbon skeleton supply (Li et al., 2025a; Vergara-Diaz et al., 2024). Compounding this metabolic constraint, crops frequently employ a foliar ion compartmentalization strategy, wherein senescing leaves preferentially sequester Na^+^ and Cl^–^. Although this mechanism transiently preserves ion homeostasis in younger leaves, it accelerates senescence in older leaves and impairs systemic nitrogen remobilization efficiency (Wang et al., 2012).

Collectively, salt-triggered C–N metabolic reprogramming embodies a short-term adaptive strategy prioritizing stress survival, with long-term physiological costs manifested as diminished NUE and biomass penalties. Given these physiological boundaries, the precise regulation of nitrogen transporters and assimilatory enzymes emerges as a critical molecular checkpoint. Thus, these mechanisms link ionic stress signaling to C–N metabolic homeostasis and mediate the transition from signal perception into the transport reprogramming and metabolic redistribution phases of the closed‑loop biological model established in this review.

## 3 Precise regulation of nitrogen transporters under salt stress: from ion uptake to long-distance allocation

The systemic reprogramming of C–N metabolism triggered by salt stress is not a passive consequence, but an actively regulated process that relies on nitrogen transporters. These transporters, governing root acquisition, intracellular compartmentalization, and long-distance translocation, act as the functional link between the “Signal Perception” and “Transport Reprogramming” phases of the closed-loop biological model proposed in this review. In this section, we focus on the nitrate/peptide transporter (NRT/NPF) and ammonium transporter (AMT) families, and examine how canonical regulatory paradigms (e.g., dual-receptor integration and kinase-mediated phosphorylation switches) regulate cellular nitrogen flux. We further examine how these local transport events influence whole-plant source-sink reprioritization under saline conditions.

### 3.1 The NRT/NPF family: from transport to sensing

The NRT/NPF family exemplifies the evolutionary convergence of transport and sensory functions, a concept pioneered by the *Arabidopsis* transceptor AtNRT1.1 (CHL1). AtNRT1.1 dynamically switches between high- and low-affinity nitrate uptake modes via phosphorylation at Thr101, while triggering downstream signaling to remodel root architecture (Ho et al., 2009). Under salt stress, however, the NRT family exhibits profound functional differentiation. The high-affinity nitrate transport system (HATS, e.g., NRT2.1/NRT2.4-NAR2.1 complexes) is generally suppressed by salinity in species such as *Arabidopsis* and wheat, reducing root nitrate influx (Muños et al., 2004). Genetic diversity exists: specific salt-tolerant rice cultivars maintain robust *OsNRT2.1* and *OsNRT2.3* expression under stress, sustaining grain yield (Li et al., 2025b).

The most striking functional divergence emerges in the signal integration strategies of low-affinity NPF members. In rice, the plasma membrane transceptor OsNRT1.1B (OsNPF6.5) has been identified as a core locus underlying subspecies nitrate utilization divergence (Hu et al., 2015). Building on its conserved nitrate-sensing function in *Arabidopsi*s (Ho et al., 2009), a recent study proposed that OsNRT1.1B may possess dual receptor activity, with structural data suggesting overlapping ABA and nitrate binding pockets (Ma et al., 2025b). This proposed mechanism requires independent genetic validation across multiple crop varieties. If validated, this activity could enable OsNRT1.1B to modulate ABA-mediated stress responses according to external nitrogen availability, providing a direct molecular link between nutrient sensing with osmotic defense (Ma et al., 2025b).

Beyond plasma membrane perception, NPF members also contribute to intracellular ion compartmentalization. Recently, the CtrNPF2.1 transporter from the woody *Citrus trifoliata* was identified as a tonoplast-localized dual-transporter. Under saline and high-nitrate conditions, the transcription factor CtrNAC019 directly activates *CtrNPF2.1*. CtrNPF2.1 then promotes vacuolar Cl^–^ sequestration (mitigating cytosolic ion toxicity) while simultaneously facilitating NO_3_^–^ efflux from the vacuole to the cytosol for assimilation (Zhao et al., 2026). This NAC–NPF regulatory module illustrates how plants decouple Cl^–^ detoxification from nitrogen utilization within a single cell type. Beyond rice and *Arabidopsis*, integrated evolutionary, structural, and transcriptional profiling of maize nitrate transporters has been performed under multiple abiotic stresses (Mia et al., 2025). Several *ZmNRT* paralogs exhibit significant transcriptional shifts under salinity stress, although functional validation of these salt-responsive candidates through transgenic knockout or overexpression remains lacking. This multi-omics dataset extends mechanistic understanding of nitrogen transport regulation.

### 3.2 The AMT family: the SOS2-AMT1;1 coupled module

As another core regulatory unit within the “Transport Reprogramming” phase, the AMT family governs ammonium uptake, a critical function given that nitrification is suppressed in salinized soils. The SOS kinase cascade is a conserved pathway balancing cellular ion homeostasis (Zhu, 2002; Shi et al., 2000). Prior work confirmed CIPK kinases negatively regulate ammonium transporter activity (Lanquar et al., 2009). Extending this framework, a recent study reports a physical interaction between SOS2 and AMT1;1 in *Arabidopsis* and proposes Ser450 activating phosphorylation to maintain ammonium uptake under salinity (Ma et al., 2025a). This kinase-transporter module has not been validated in cereal species.

Crucially, this ammonium uptake establishes a positive feedback loop by further enhancing SOS2 kinase activity, which concurrently maintains cytosolic K^+^/Na^+^ homeostasis via canonical SOS pathway effectors (Ma et al., 2025a). This activating phosphorylation by SOS2 is counterbalanced by the CIPK23-dependent phosphorylation of the inhibitory Thr460 residue on AMT1;1 (Lanquar et al., 2009). Together, these opposing kinase activities constitute a rheostat: preventing cytotoxicity from excessive cytosolic ammonium accumulation while securing sufficient nitrogen flux for survival under saline duress.

The regulatory patterns of the AMT family also exhibit species specificity. The rice OsAMT1 family comprises three members (OsAMT1;1, OsAMT1;2, and OsAMT1;3), which collectively mediate root ammonium uptake under diverse nitrogen conditions (Konishi and Ma, 2021). It is well established that NH_4_^+^ and Cl^–^ are taken up synergistically under salt stress, whereas NO_3_^–^ competes with Cl^–^ for transporter binding sites; as a result, sole ammonium nutrition typically exacerbates salt stress damage in most species (Liu et al., 2020). However, exceptions exist: in maize, ammonium nutrition supports better growth performance than nitrate nutrition under osmotic stress, highlighting the species-dependent nature of nitrogen-form adaptation (Hessini et al., 2019). This dictates that mechanism-driven breeding must target crop-specific AMT regulatory networks rather than blindly extrapolating from model dicots.

### 3.3 Systemic integration: source–sink reprioritization under salt stress

Local transport events at the root interface are ultimately integrated into systemic nitrogen allocation. Salt stress resets the whole-plant source-sink priority: nitrogen is diverted away from structural anabolism in sink tissues toward the biosynthesis of amino acid-derived osmoprotectants in mature source leaves (Vergara-Diaz et al., 2024).

This systemic reprioritization is achieved through the coordinated control of xylem loading and phloem retrieval. In *Arabidopsis*, the antagonism between NRT1.5 (responsible for root-to-shoot xylem loading) and NRT1.8 (mediating xylem retrieval) leads to preferential nitrate retention in roots under salt stress. This “shoot nitrogen starvation” serves as an adaptive strategy to restrict vegetative growth and fortify root stress defenses (Chen et al., 2012). Concurrently, salt-stressed senescing leaves undergo a metabolic shift from the efficient GS-GOGAT cycle to the GDH shunt, impairing whole-plant nitrogen remobilization (Wang et al., 2012). Thus, nitrogen transporters not only execute ion flux but enforce the growth-defense trade-off, serving as the functional link between transport reprogramming and downstream metabolic redistribution (**Table 1**).

**Table 1:** Core regulatory mechanisms of nitrogen transporters under salt stress.

Transporter	Organism	Substrate	Salt stress response	Regulatory mechanism	Functional outcome	References
*NRT1.1*/*NPF6.3*	*Arabidopsis*	NO_3_^–^	Phosphorylation-dependent activity modulation	Thr101 phosphorylation toggles nitrate affinity; mediates nitrate-triggered Ca^2+^ signaling and root architecture remodeling	Balances high/low-affinity nitrate uptake; coordinates root development under salinity	Ho et al., 2009; Liu and Tsay, 2003
*OsNRT1.1B*/*OsNPF6.5*	*Oryza sativa*	NO_3_^–^	Natural variation alters salt tolerance and NUE	Functions as dual receptor for nitrate and ABA; competitively binds the same ligand pocket and interacts with OsSPX4 to release OsNLP4	Integrates nutrient and stress signaling; serves as core target for synergistic crop improvement	Ma et al., 2025b; Hu et al., 2015
*NRT2.1*	*Arabidopsis*	NO_3_^–^	Transcriptionally downregulated	Requires NAR2.1 as co-factor; transcriptionally activated by NLP7 under sufficient nitrate	Reduces high-affinity nitrate uptake under high salinity	Muños et al., 2004; Liu et al., 2017
*OsNRT2.1*/*OsNRT2.3*	*Oryza sativa* (SR86)	NO_3_^–^	Expression maintained or upregulated	Upstream transcriptional regulatory mechanism remains largely uncharacterized	Sustains root nitrate uptake and long-distance root-to-shoot translocation under salt stress	Li et al., 2025b
*AMT1;1*	*Arabidopsis*	NH_4_^+^	Bidirectional activity modulation	SOS2 phosphorylates Ser450 for activation; CIPK23 phosphorylates Thr460 for inhibition	Balances ammonium acquisition and cytotoxicity avoidance; couples ion homeostasis	Lanquar et al., 2009; Ma et al., 2025a
*CtrNPF2.1*	*Citrus trifoliata*	NO_3_^–^/Cl^–^	Induced by both salt stress and high nitrate	Directly activated by transcription factor CtrNAC019; exhibits pH-dependent bidirectional transport activity	Sequesters Cl^–^ into vacuoles for detoxification; remobilizes vacuolar nitrate for assimilation	Zhao et al., 2026
*SsNRT2.5*	*Suaeda salsa*	NO_3_^–^	Strongly induced by salt stress	Native promoter harbors salt-responsive and nitrogen metabolism cis-elements	Enhances nitrate accumulation and salt tolerance under saline conditions	Liu et al., 2026b

## 4 Enzymatic regulation of nitrogen assimilation and the carbon–nitrogen coupling under salt stress

Following root nitrogen uptake, inorganic nitrogen must be assimilated into organic forms to support cellular biosynthesis. However, salt stress impairs photosynthetic carbon fixation, resulting in a deficiency of intracellular carbon skeletons, which represents the primary metabolic bottleneck for nitrogen assimilation. This section examines the regulation of key assimilatory enzymes under salt stress and the metabolic partitioning strategies triggered by carbon limitation, thereby clarifying the biochemical nature of the growth-defense trade-off.

### 4.1 NR and NiR: nitrate reduction and its metabolic crosstalk with nitric oxide signaling

NR serves as the rate-limiting enzyme in the nitrogen assimilation pathway, exerting dual roles in catalytic metabolism and signal transduction under salt stress. Genetic evidence from rice *nr1/nr2/nr3* triple mutants demonstrates that loss of NR activity enhances salt sensitivity under nitrate nutrition. Crucially, this phenotype can be fully rescued by exogenous application of NO donors, indicating that NR-dependent endogenous NO production is a key mediator of nitrate-promoted salt tolerance (Teng et al., 2025).

NR-derived NO functions as a signaling molecule that modulates antioxidant defenses and ion transport via protein *S*‑nitrosylation, thereby alleviating salt-induced oxidative damage (Liu et al., 2026a). The repression of NR activity under salt stress is not merely a passive consequence of injury but an actively regulated response. Under energy or carbon deficits, NR is phosphorylated at specific serine residues (e.g., Ser543 in spinach), promoting the binding of inhibitory 14-3–3 proteins. This conformational change blocks electron transport, thereby inactivating the enzyme (Bachmann et al., 1996; Kaiser and Huber, 2001). This rapid regulatory switch enables plants to adjust nitrogen assimilation flux according to cellular energy status.

Concurrently, nitrite reductase (NiR) activity is coordinately downregulated alongside NR. The magnitude of NiR inhibition under salinity is constrained by carbon skeleton availability, as suppressed photosynthesis restricts the supply of reductants (NADH) and 2‑oxoglutarate (2‑OG), disrupting the coordinated operation of nitrate and nitrite reduction (Debouba et al., 2006).

### 4.2 The GS-GOGAT cycle and GDH shunting: metabolic acclimation under carbon skeleton limitation

Severe limitation of carbon skeleton availability triggers a fundamental metabolic repartitioning in nitrogen assimilation. The GS‑GOGAT cycle serves as the primary ammonium assimilation pathway; however, due to its high demand for ATP and 2‑OG, its catalytic efficiency is constrained by carbon supply. Consequently, salt‑induced carbon scarcity broadly represses the operation of the GS‑GOGAT cycle (Nazir et al., 2023).

Tissue-specific response patterns underpin this metabolic reprogramming. In rice, salt stress strongly represses the expression of the plastid isoform *OsGS2* and ferredoxin dependent *OsFd-GOGAT* in senescent leaves, while cytosolic *OsGS1* family members (*OsGS1;1*, *OsGS1;2*, *OsGS1;3*) are markedly upregulated (Wang et al., 2012). This enzymatic divergence is linked to the accumulation of excess Na^+^ and Cl^–^ in senescent leaves: ion toxicity disrupts chloroplast integrity and suppresses photorespiration, thereby diminishing the photorespiratory NH_4_^+^ pool on which *OsGS2* and *OsFd-GOGAT* depend.

Mechanistically, this disruption of organellar integrity directly curtails compartmentalized nitrogen fluxes. Salt‑induced thylakoid swelling and chloroplast envelope destruction, associated with ionic and osmotic stress, respectively, have been documented by transmission electron microscopy in rice (Yamane et al., 2003). These ultrastructural lesions impair photophosphorylation and restrict the cellular supply of ATP and reduced ferredoxin, suppressing the catalytic capacity of plastidic GS2/Fd‑GOGAT (Wang et al., 2012). Concurrently, salt‑triggered oxidative damage impairs mitochondrial nitrogen recycling. The lipid peroxidation product 4‑hydroxy‑2‑nonenal (HNE) inhibits glycine decarboxylase complex (GDC) H‑protein activity by modifying its lipoic acid moiety (Taylor et al., 2002), curtailing photorespiratory NH_4_^+^ release and further depleting the substrate pool for chloroplastic GS2. These organellar constraints are initiated by upstream ROS–Ca^2+^ signaling cascades.

As a consequence of this organellar impairment, transcript levels of *OsGDH2* and *OsGDH3* are significantly elevated in salt‑stressed senescent leaves, suggesting a partial shift of nitrogen flux toward the glutamate dehydrogenase (GDH) pathway to sustain basal glutamate homeostasis (Wang et al., 2012). This reciprocal shift between the GS‑GOGAT and GDH pathways reflects a tissue‑specific acclimation to carbon and energy deficits, albeit at the cost of reduced overall NUE. A quantitative comparison of core carbon‑nitrogen metabolic pathways in terms of energy cost, stress response, and regulatory hubs is provided in **Table 2**, summarizing the metabolic basis of the growth‑defense trade‑off under salinity.

**Table 2:** Regulation and adaptation of key carbon–nitrogen metabolic pathways under salt stress.

Metabolic pathway	Core enzyme	Carbon substrate	Nitrogen product	Salt-induced change	Functional trade-off	Regulatory hub	References
Nitrate reduction pathway	NR, NiR	No direct carbon skeleton; requires reducing power (NADH/Fdred) from carbon metabolism	NH_4_^+^ (via NO_2_^–^ intermediate)	↓ Significantly downregulated	Drives core nitrogen assimilation and NO-mediated stress defense; highly dependent on carbon-derived reducing equivalents	SnRK1 (inhibitory); NLP transcription factors (activatory)	Kaiser and Huber, 2001; Debouba et al., 2006
GS-GOGAT cycle	GS1, GS2, Fd-GOGAT, NADH-GOGAT	2-oxoglutarate (2-OG)	Glutamate (net product)	↓ Reduced metabolic flux	High nitrogen assimilation efficiency; strictly dependent on ATP and carbon skeleton supply	TOR kinase (activatory); SnRK1 (inhibitory)	Wang et al., 2012; Nazir et al., 2023
GDH pathway	GDH1, GDH2, GDH3	2-OG	Glutamate (synthetic direction dominates under salt stress)	↑ Activated under severe salt stress	Low assimilation efficiency; serves as emergency ammonium detoxification bypass	SnRK1 (energy-sensing)	Wang et al., 2012
Proline biosynthesis pathway	P5CS1/2, P5CR, ProDH	Glutamate	Proline	↑↑ Strongly induced	Prioritizes osmotic adjustment for stress survival; competes with primary assimilation for carbon skeletons	ABA-dependent transcriptional regulation of *P5CS1*	Carillo et al., 2008; Liu et al., 2024
Asparagine synthesis pathway	AS1, AS2	Aspartate	Asparagine	↑ Induced for nitrogen storage	Enables stable long-distance nitrogen transport and storage; low bioavailability for rapid growth	NLP transcription factors	Meng et al., 2016

### 4.3 Transcriptional and metabolic reprogramming of nitrogen networks

Under salt stress, crops undergo genome-wide transcriptional and metabolic reprogramming that redirects nitrogen flux from primary anabolic assimilation toward stress‑defense metabolism.

Transcriptomic and proteomic evidence supports this global shift. Salinity represses core primary nitrogen assimilation genes (e.g., *OsGS2*, *OsFd-GOGAT*) while upregulating GDH family genes (Xu et al., 2016). Salt stress reduces the abundance of the PII nitrogen‑sensing protein and suppresses transcripts encoding multiple ribosomal subunits in leaves. This restriction of cellular protein synthesis diverts surplus amino acids toward the biosynthesis of compatible solutes (e.g., proline and glycine betaine), thereby linking nitrogen metabolism to osmotic stress defense (Carillo et al., 2008; Xu et al., 2016).

The global reprogramming of nitrogen assimilation constitutes the fundamental metabolic foundation underlying the growth‑defense trade‑off. Three regulatory modalities collectively enable the redistribution of nitrogen for stress survival: post‑translational modification of key enzymes (e.g., NR phosphorylation), tissue‑specific metabolic shunting (from GS‑GOGAT to GDH), and genome‑wide transcriptional and translational restriction. The upstream signaling drivers and transcription factors coordinating this metabolic shift will be examined in the following section.

## 5 Signaling networks and transcriptional hierarchies underlying salt-nitrogen crosstalk

Plants have evolved a hierarchically organized molecular cascade from membrane perception to nuclear reprogramming, enabling the precise integration of salt stress and nitrogen signals. This cascade serves as a signal-integration hub in the closed-loop biological model, linking upstream sensory/transport modules to downstream metabolic acclimation. This section examines interconnected regulatory tiers, including ROS–Ca^2+^–MAPK-mediated perception and amplification, rapid regulation by PTM, including phosphorylation and *S*‑nitrosylation and phytohormonal coordination. It also considers transcriptional regulation by NLP and NAC transcription factors, as well as TOR–SnRK1-mediated metabolic homeostasis.

### 5.1 The ROS-Ca^2+^-MAPK cascade: early salt stress perception and signal amplification

Salt stress signal initiation stems from the coordinated detection of ionic toxicity and osmotic stress at the plasma membrane. Glycosyl inositol phosphorylceramide (GIPC) sphingolipids function as specific Na^+^ sensors, triggering cytosolic Ca^2+^ transients via MOCA1-dependent Ca^2+^ channels (Jiang et al., 2019). In parallel, the osmosensitive channel OSCA1 perceives turgor fluctuations, together defining the initial Ca^2+^ signature (Yuan et al., 2014). This salt-elicited Ca^2+^ surge activates NADPH oxidases (RBOHD/F), triggering a burst of ROS that forms a self-amplifying feedback loop to propagate the stress signal (Gong et al., 2020; Yang and Guo, 2018). Amplified Ca^2+^–ROS signals are then transduced to MAPK cascades. Two core axes dominate salt stress signaling: MEKK1–MKK1/2–MPK4 and MKK4/5–MPK3/6, both of which relay signals to the nucleus through sequential phosphorylation events (Yang and Guo, 2018).

The integration of salt-elicited Ca^2+^ waves and nitrate-specific Ca^2+^signatures constitutes the core node of early crosstalk. Nitrate elicits distinct Ca^2+^ signatures via the NRT1.1-CNGC15 receptor-channel complex, promoting the nuclear translocation of NODULE INCEPTION-LIKE PROTEIN (NLP) transcription factors (Wang et al., 2021a). In rice, OsCNGC14 and OsCNGC16 function as downstream Ca^2+^ channels. Under saline conditions, excess Na^+^ inhibits their channel activity, altering the amplitude and duration of nitrate-evoked Ca^2+^ transients, thereby compromising NLP nuclear localization and downstream gene expression (Wang et al., 2025; Qu et al., 2026). These early signals are transduced through MAPK cascades and PTM networks to modulate downstream nitrogen metabolism.

### 5.2 Post‑translational modification hubs: phosphorylation and *S*‑nitrosylation

PTMs constitute the fastest-acting regulatory layer, allowing crops to dynamically remodel nitrogen metabolism within minutes. Phosphorylation governs this crosstalk through classical paradigms. The salt-activated kinase SOS2 directly phosphorylates AMT1;1 at Ser450 to sustain ammonium uptake, whereas CIPK23-mediated phosphorylation at Thr460 inhibits AMT1;1 to prevent toxicity (Lanquar et al., 2009; Ma et al., 2025a). Concurrently, phosphorylation of NRT1.1 at Thr101 toggles its nitrate affinity state, linking nutrient perception to intracellular signal transduction (Liu and Tsay, 2003). Notably, this phosphorylation switch not only regulates transport activity but also triggers calcium-mediated downstream signaling.

*S*‑nitrosylation exerts bidirectional effects. Under salt stress, NR-dependent NO generation triggers *S-*nitrosylation that enhances proline biosynthesis via P5CR activation (Liu et al., 2024), while repressing H^+^-ATPase2 activity to reduce the proton motive force driving nitrogen uptake (Wei et al., 2025). Furthermore, *S*‑nitrosylation of the receptor-like kinases SERK3A and SERK3B activates brassinosteroid signaling, extending the regulatory repertoire of this PTM in crop salt tolerance (Wei et al., 2024b).

Cross-regulation between phosphorylation and *S*‑nitrosylation remains underexplored. Whether SOS2 kinase activity is itself regulated by *S*‑nitrosylation, or whether NLP transcription factors are subject to dual phosphorylation and *S*‑nitrosylation control, remain priority research questions. The synergistic action of these two PTM systems forms the core regulatory network for salt-nitrogen crosstalk, mediating rapid and precise reprogramming of nitrogen metabolism in response to saline stress.

### 5.3 Hormone signaling crosstalk: ABA, ethylene, and auxin

Phytohormone networks integrate salt stress and nitrogen status signals, coordinating stress responses across tissues and developmental stages. In rice, OsNRT1.1B has been hypothesized to act as a dual receptor for nitrate and ABA (Ma et al., 2025b). Upon ABA binding, OsNRT1.1B interacts with OsSPX4, leading to the release of the transcription factor OsNLP4 and activation of ABA-responsive gene expression. This interaction enables receptor-level signal integration (Ma et al., 2025b).

Additional phytohormone pathways regulate distinct components of this response. Under salt stress, transcriptional suppression of NRT1.5 promotes nitrate retention in roots to improve stress resilience (Chen et al., 2012). Concurrently, ethylene signaling modulates nitrogen allocation and ion homeostasis in a nitrogen-form-dependent manner by regulating NRT1.5 transcript levels, thereby linking long-distance nitrate transport and root-to-shoot K^+^ distribution (Chen et al., 2021). Auxin modulates nitrogen foraging by remodeling root architecture via NRT1.1-mediated auxin flux (Krouk et al., 2010). These pathways coordinate nitrogen transport, allocation and salt stress responses through receptor-level signal integration and downstream transcriptional regulation, supporting whole-plant stress acclimation.

### 5.4 Transcriptional execution hubs: NLP, NAC, bZIP, and MYB

Transcription factors integrate upstream signals to reprogram nitrogen metabolism. The NLP family comprises conserved master regulators of nitrogen signaling in plants. In *Arabidopsis*, nitrate‑induced Ca^2+^ signaling triggers the nuclear translocation of NLP7 (Liu et al., 2017). In rice, *OsNLP4* directly binds nitrate‑responsive *cis*‑elements (NREs) to activate target genes, including *OsNiR* (Yu et al., 2021), whereas phosphorylation of *OsNLP3* at Ser193 promotes its nuclear translocation (Wang et al., 2025). Salt stress also upregulates *OsNLP2*, increasing *OsNR1/2* transcript levels and NR activity, while promoting ABA catabolism to enhance salt tolerance during germination (Yi et al., 2022).

NLP activity is regulated by ubiquitination pathways. In rice, the E3 ubiquitin ligase NBIP1, which is recruited by OsNRT1.1B, mediates OsSPX4 degradation and releases OsNLP3 for nuclear translocation (Ma et al., 2025b). In *Arabidopsis*, the E3 ligase HOS1 (high expression of osmotically responsive genes 1) regulates nitrate signaling by modulating the nuclear import and turnover of SnRK1α1, thereby influencing NLP7 nuclear trafficking and stability (Xie et al., 2026). Beyond NLPs, several transcriptional modules coordinate nitrogen metabolism with ion homeostasis under salt stress. The CtrNAC019-CtrNPF2.1 module coordinates ion detoxification and nitrogen remobilization (Zhao et al., 2026), OsMYB2 activates the amino acid transporter *OsANT1* to promote resource redistribution (Nie et al., 2025). OsbZIP72 upregulates *OsHKT1;1* to maintain K^+^/Na^+^ homeostasis, thereby indirectly supporting nitrogen metabolism under salt stress (Wang et al., 2021b). These regulatory pathways contribute to the balance between growth and stress acclimation and may improve nitrogen use efficiency under saline conditions.

The downstream targets of these transcription factors include genes encoding membrane sensors, nutrient transporters, assimilatory enzymes, and signaling kinases. These interactions form a signal–transcription–feedback regulatory loop that contributes to the maintenance of cellular nitrogen homeostasis under salt stress.

### 5.5 Energy and metabolic sensing: the TOR-SnRK1 module

Salt stress represses photosynthetic carbon fixation, resulting in insufficient ATP and NADPH production and a cellular energy deficit. The evolutionarily conserved TOR and SnRK1 pathways act as global sensors of carbon and nitrogen status and integrate energy homeostasis with salt–nitrogen crosstalk (Artins et al., 2024; Xu et al., 2025). Under energy‑replete conditions, TOR promotes protein synthesis and cell growth, thereby sustaining anabolic metabolism. Salt stress‑induced energy deprivation inhibits TOR activity and activates SnRK1, initiating stress acclimation programs.

SnRK1 regulates nitrogen metabolism at multiple levels. It phosphorylates the nitrate channel SLAH3 to alleviate ammonium toxicity (Sun et al., 2021), and phosphorylates NR to suppress catalytic activity and conserve energy (Sugden et al., 1999). At the transcriptional level, SnRK1 promotes NLP7 degradation, thereby repressing nitrogen-responsive gene expression (Wang et al., 2022), and activates bZIP63 to redirect nitrogen resources from protein synthesis toward alternative energy production (Artins et al., 2024). In addition, SnRK1 induces autophagy, which recycles cellular components and release amino acids for stress acclimation (Chen et al., 2017). Although this metabolic reprogramming enhances short‑term salt tolerance, it can deplete carbon skeletons and sequester nitrogen in defensive metabolites, ultimately reducing grain yield and nitrogen use efficiency. The TOR–SnRK1 module links metabolic acclimation with signal integration by coupling cellular energy status to nitrogen metabolic decisions. It is therefore an important regulator of the growth–defense trade-off and a potential genetic target for mitigating the negative association between salt tolerance and high yield through genome editing and precision breeding.

Salt-nitrogen crosstalk is regulated across several cellular levels. Stress perception begins with ROS-Ca^2+^ signaling at the plasma membrane, followed by rapid post-translational modifications in the cytoplasm and transcriptional reprogramming in the nucleus. The TOR–SnRK1 module coordinates these responses with cellular energy status and metabolic acclimation. Core regulatory nodes, including OsNRT1.1B, SOS2, NLP7, SnRK1 and HOS1, provide mechanistic entry points for investigating salt–nitrogen interactions and potential targets for improving salt tolerance and nitrogen use efficiency through precision breeding.

## 6 Genetic dissection of salt‑tolerant and high nitrogen use efficiency traits: from natural variation to breeding targets

Building upon the signaling networks delineated, this chapter examines genetic variation as a bridge between upstream molecular mechanisms and downstream breeding applications within the closed-loop biological model. NUE under salt stress is a complex quantitative trait controlled by multiple loci and strongly influenced by G×E interactions. Its regulation involves coordinated nitrogen assimilation, ion homeostasis, and osmotic adjustment. Advances in QTL mapping, GWAS, wild germplasm mining, and genomic selection have enabled the systematic dissection of the genetic architecture underlying these traits, uncovering elite natural alleles that serve as direct targets for mechanism-driven crop design.

### 6.1 Genetic architecture of NUE remodeled by salt stress: from QTL mapping to GWAS dissection

Early QTL analyses of NUE were largely performed under single nitrogen regimes without salt treatment, yielding loci with limited relevance to salinized agricultural systems. Agrama et al. (1999) mapped the first set of NUE-related QTLs in maize, but the absence of salt stress in the experimental design made the identified loci difficult to deploy in saline-alkali environments. Subsequent multi-environment trials in barley explicitly differentiated low-nitrogen stress from combined salt-nitrogen stress, identifying candidate loci encoding nitrogen transporters (NRTs, AMTs) and assimilatory enzymes (GSs, GOGATs) associated with combined salt and nitrogen stress (Han et al., 2016).

This salt-specific remodeling of the NUE genetic architecture has been supported by large-scale GWAS. Phan et al. (2023b) profiled a panel of 2,391 rice accessions treated with 60 mM NaCl under normal and low-nitrogen conditions, identifying 55 genetic loci significantly associated with NUE. Only three QTLs detected under standard nitrogen supply co-localized across salt-stressed and non-saline environments, whereas the vast majority represented salt-stress-specific genomic segments. No single QTL was detectable across all four nitrogen-salt combinations. This finding suggests that salt stress is associated with a distinct genetic regulatory program. Dissecting this stress-specific network is important for breeding crops with both salt tolerance and high NUE.

Meta-analysis has emerged as a robust strategy to address the low reproducibility of single-study QTLs arising from G×E interactions. In wheat, Saini et al. (2021) integrated 1,788 QTLs from 24 independent studies to identify 118 meta-QTLs, 88 of which were validated by GWAS. The confidence intervals of these meta-QTLs were approximately nine-fold narrower than those of the original QTLs, thereby improving mapping resolution and reducing false positives associated with environmental heterogeneity. Nevertheless, G×E interaction remains a major constraint on translational breeding: QTLs with large effect sizes under controlled greenhouse conditions often exhibit attenuated or even opposite effects in field environments characterized by spatially variable salinity and dynamic nitrogen availability. Therefore, establishing standardized multi-environment phenotyping datasets, that encompass diverse salinity gradients and nitrogen levels, together with multi-environment trial GWAS (MET-GWAS) is essential for evaluating the breeding utility of candidate loci.

### 6.2 Functional validation of core genes governing salt-nitrogen coordination

Coupling genetic mapping with transcriptomic profiling and reverse genetics has enabled the cloning of key functional genes mediating NUE under salt stress. The following three paradigmatic cases, representing distinct regulatory modes (root xylem loading, amino acid metabolism, and vacuolar compartmentalization), highlight the mechanistic diversity of elite alleles.

(1) Maize *NCR1-NRT2.3* module: a regulator of root nitrate allocation under stress.

Zhang et al. (2026a) identified a major QTL associated with xylem nitrate concentration in maize roots via GWAS and confirmed *NCR1*, encoding an R2R3-MYB transcription factor, as the causal gene. Root-specific *NCR1* harbors a 123-bp insertion/deletion (InDel) in its promoter defining two major haplotypes. Accessions carrying the deletion allele (*NCR1^–In^*) exhibit significantly higher xylem nitrate content under low nitrogen, indicating that this InDel is the functional variant driving differential transcription. Mechanistically, NCR1 directly binds to the promoter of *NRT2.3*, activating its expression in xylem parenchyma cells to mediate nitrate loading for long-distance transport to shoots. Overexpression of either *NCR1* or *NRT2.3* significantly improves grain yield and NUE in maize under low-nitrogen conditions. Notably, this elite allele was gradually lost during modern maize breeding under intensive nitrogen fertilization, illustrating the potential to recover elite alleles lost during domestication.

(2) Rice *OsLHT5*: an amino acid transporter linking nitrogen assimilation and osmotic tolerance.

Wang et al. (2026) identified OsLHT5, encoding a lysine-histidine type transporter, as a significant locus associated with effective panicle number via rice GWAS. Haplotype analysis revealed that *LHT5^HapA^*, carrying a 30 bp deletion in the coding region, is an elite allele. Overexpression of *LHT5^HapA^* significantly increases accumulation of neutral amino acids (glycine and threonine) and upregulates the transcription of *OsP5CS1/2*, key genes for proline biosynthesis. Under combined low-nitrogen and salt stress, *LHT5^HapA^* overexpression lines show both enhanced nitrogen accumulation and salt tolerance, supporting a role for amino acid metabolism in coordinating NUE and salt tolerance. This elite allele is mainly found in wild rice and landraces (distribution frequency <5% in modern cultivars), indicating the breeding potential of wild germplasm resources.

(3) *Citrus trifoliata CtrNAC019-CtrNPF2.1* module: vacuolar compartmentalization for ion and nitrogen coordination.

In *Citrus trifoliata*, Zhao et al. (2026) identified a regulatory module induced by both salt and high nitrate. The transcription factor CtrNAC019 directly activates *CtrNPF2.1*, which encodes a tonoplast-localized nitrate transporter. CtrNPF2.1 exhibits pH-dependent bidirectional transport activity: it sequesters Cl^–^ into vacuoles for ion detoxification under salt stress and exports vacuolar NO_3_^–^ into the cytosol for assimilation when nitrogen is sufficient. This module illustrates the coordination of salt tolerance and NUE at the organellar level and may provide a target for organelle engineering to improve crop performance under saline conditions.

### 6.3 Limitations of marker-assisted selection and opportunities for genomic selection

Translating elite alleles into actual breeding gains relies on an efficient molecular breeding system. Traditional marker-assisted selection (MAS) has inherent limitations for complex quantitative traits such as NUE: it tracks only a limited number of major-effect genes, whereas NUE is controlled by numerous minor-effect loci with pervasive epistatic interactions. Consequently, MAS is inefficient at pyramiding multiple salt-tolerant and nitrogen-efficient loci and lacks the capacity to accommodate phenotypic variation arising from G×E interactions (Hu et al., 2023).

Genomic selection (GS) offers an alternative approach to address this gap. By training prediction models using genome-wide, high-density markers and phenotyped reference populations, GS captures both major and minor genetic effects, enabling the accurate prediction of genomic estimated breeding values (GEBVs). In maize and wheat breeding programs targeting combined salt stress and low nitrogen, GS has been shown to achieve higher prediction accuracy for NUE than conventional MAS (Budhlakoti et al., 2022). Nevertheless, two key challenges restrict GS application in saline environments. First, most training populations are dominated by conventional cultivars, with limited representation of salt-adapted germplasm and rare elite alleles identified from natural variation. Second, modeling G×E interactions under combined salt-nitrogen stress requires large-scale, multi-environment phenotypic data and environmental covariates, which remain scarce for most crop species. Elite natural alleles identified via GWAS (e.g., *NCR1*, *OsLHT5*) can be integrated into GS models as fixed-effect markers to potentially improve prediction accuracy. Future breeding frameworks could integrate MAS and GS: fixing validated major elite alleles via MAS while pyramiding numerous minor favorable loci via GS, thereby achieving mechanism-driven improvement of salt-tolerant and nitrogen-efficient crops.

## 7 Genome editing and design breeding: from precise molecular engineering to synthetic biology

The preceding chapter summarized naturally occurring elite alleles identified via QTL mapping and genome-wide association studies (GWAS) (e.g., maize *NCR1*, rice *OsLHT5*, and trifoliate orange *CtrNPF2.1*), which provide valuable targets for crop molecular breeding. However, natural evolution proceeds slowly, and elite alleles are frequently linked to unfavorable agronomic traits because of linkage drag, limiting their direct introgression into modern breeding programs (Padmavathi et al., 2024). Precision genome editing technologies, particularly CRISPR/Cas9 and its derivative tools, have shifted breeding strategies from passive “discovery–utilization” to active “design–creation” approaches. This chapter examines the translational application of the salt–nitrogen regulatory framework by integrating recent advances in genome editing and synthetic biology. We focus on how these tools can be used to modify validated targets and construct autonomous regulatory circuits for simultaneously improving salt tolerance and NUE.

### 7.1 Precise single-gene editing: from functional validation to targeted trait improvement

Single-gene editing represents an established precision breeding strategy, achieving targeted trait optimization through three approaches: elite allele replacement, negative regulator knockout, and *cis*-regulatory element modification.

Elite allele replacement enables the direct introduction of favorable natural variants into elite genetic backgrounds, thereby bypassing linkage drag. In rice, Li et al. (2018) precisely introgressed the elite *indica OsNRT1.1B* allele into a *japonica* background via CRISPR/Cas9-mediated homologous recombination, resulting in significantly improved nitrate uptake efficiency under low-nitrogen conditions. As discussed above, OsNRT1.1B has been proposed to function as a dual-function transceptor that coordinates nutrient and ABA signals at the plasma membrane; however, this regulatory mechanism requires independent verification (Ma et al., 2025b). This proposed coupling makes OsNRT1.1B a promising target for the coordinated improvement of NUE and osmotic adaptation.

The targeted knockout of negative regulators offers another effective strategy.*ARE1* is a characterized negative regulator of NUE in rice; its CRISPR/Cas9-mediated knockout enhances nitrogen assimilation under low-nitrogen conditions, increasing grain yield by 10%–20% with no detectable agronomic trade-offs (Wang et al., 2021c). Similarly, the targeted disruption of *OsRR22* markedly enhances salt tolerance in rice without compromising major yield components (Sheng et al., 2023).

Furthermore, base editing and prime editing enable precise single-nucleotide substitutions, providing high-resolution tools for modifying functional amino acid residues (Fiaz et al., 2021). For instance, Thr327Met natural variant of rice OsNRT1.1B has been shown to enhance NUE (Hu et al., 2015). Cytosine base editing systems have been demonstrated to specifically target this coding locus, providing a viable approach for the *de novo* generation of this elite variant (Lu and Zhu, 2017). In addition, the CRISPR/dCas9-mediated activation (CRISPRa) system can upregulate target gene expression in a graded manner without altering genomic sequences, providing a flexible means for optimizing the salt-nitrogen crosstalk network (Shelake et al., 2022).

### 7.2 Multigene editing: targeted pyramiding of salt tolerance and high NUE traits

NUE is a complex quantitative trait, and manipulation of a single gene often fails to achieve robust field performance under severe salt stress. Multiplex CRISPR/Cas9 systems, such as tRNA–gRNA arrays, enable the simultaneous targeting of multiple genomic loci in a single transformation event, providing a technical basis for the simultaneous pyramiding of multiple traits (Ma et al., 2015; Xu et al., 2019).

A representative application was reported by Hao et al. (2025), who used multiplex CRISPR/Cas9 editing to modify 13 agronomically important genes in the salt tolerant rice germplasm SR86. Through sequential transformation and progeny selection, they developed the improved line SR86M, which carried nine desired functional mutations. This strategy followed a modular logic: *ARE1* knockout enhanced NUE; mutations in *SD1* and *OTUB1* optimized canopy architecture and lodging resistance; combined mutations in *GS3*, *GW8*, *GS9*, and *Gn1a* improved grain yield potential; and mutations in *Hd1* and *BADH* attenuated photoperiod sensitivity and conferred fragrance. Salt tolerance assays showed that SR86M maintained survival rates under 1.2% NaCl stress comparable to those of wild-type SR86 and significantly higher than those of conventional cultivars. This study demonstrates the potential of multiplex editing for the coordinated improvement of multiple traits under saline conditions.

Despite its potential, multiplex editing faces bottlenecks, including off-target mutations and antagonistic pleiotropy among target pathways. The use of high-fidelity Cas9 variants combined with optimized sgRNA design significantly mitigates off-target risks (Xu et al., 2019). However, regulatory frameworks differ among countries and regions: China implements safety certification management for gene‑edited crops, the EU categorizes most edited materials as GMOs subject to stringent regulation, and the United States adopts product-oriented regulatory oversight. In addition to regulatory differences, regional variation in public acceptance and the high costs associated with multi-year variety registration remain major barriers to field-scale application. Beyond multiplex editing, newer tools, including base editing (Ren et al., 2021), prime editing, and CRISPR interference (CRISPRi) (Zeng et al., 2020; Xue et al., 2023), enable finer modulation of nitrogen metabolism genes, and complement the knockout-based strategies described above. Integrating these multigene editing platforms with traditional marker-assisted selection may provide an effective breeding framework (Neeraja et al., 2021), thereby facilitating the transition from single-gene modification to the coordinated engineering of complex traits.

### 7.3 Synthetic biology-driven regulatory circuits

Synthetic biology offers approaches to address the inherent physiological constraints on nitrogen utilization under salt stress through the rational design of artificial genetic circuits. These circuits are built on signaling networks discussed in earlier sections, including NRT1.1-mediated perception, ABA signaling, and ROS–Ca^2+^ cascades. This section examines four research directions; the first three may have translational potential, whereas artificial nitrogen fixation remains an exploratory goal.

#### 7.3.1 Engineered nitrogen transporters

Structure-guided and deep-learning-assisted protein design can be used to modify the substrate specificity of existing transporters. Under salt stress, high extracellular Cl^–^ competitively interferes with NO_3_^–^ binding to transporters, thereby restricting nitrogen uptake (Liu et al., 2020). The resolution of NPF-family crystal structures, including that of NRT1.1, provides a structural basis for site-directed mutagenesis aimed at enhancing nitrate selectivity while reducing chloride affinity (Sun et al., 2014). Although AI-assisted protein design may accelerate this process, the field remains at the *in planta* proof-of-concept stage.

#### 7.3.2 Salt stress-inducible orthogonal regulatory circuits

Using salt-specific promoters, such as *RD29*A and *SOS1*, to drive the expression of nitrogen transporters could create an autonomous “stress perception → enhanced nitrogen response” circuit, thereby avoiding the metabolic costs of constitutive overexpression. Halophytes may provide useful sources of such promoters. Recently, Liu et al. (2026b) cloned the *SsNRT2.5* and its native promoter from *Suaeda salsa*, which is enriched with stress-responsive (GT-1, DRE) and nitrogen-responsive (GATABOX) *cis-*elements. In transgenic *Arabidopsis* and rice, *SsNRT2.5* driven by this native promoter facilitated higher NO_3_^–^ accumulation, reduced Na^+^ content, and significantly increased seed nitrate storage and grain weight under salt stress. Coupling the OsNRT1.1B transceptor with an artificial transcriptional activation system could establish a closed-loop “nitrate/salt concentration → calcium signature → transcriptional output” regulatory platform.

#### 7.3.3 Engineered carbon–nitrogen metabolic flux

Insufficient carbon skeleton supply under salt stress is a major constraint on nitrogen-assimilation efficiency. Tissue-specific expression of key carbon-metabolism enzymes, such as phosphoenolpyruvate carboxylase, can increase carbon-skeleton availability in roots. CRISPRi-mediated downregulation of the GDH pathway could theoretically stabilize GS–GOGAT cycle flux. However, this strategy would need to avoid excessive TOR–SnRK1-mediated energy-conservation responses, as discussed in Section 5. These dynamic rewiring strategies require quantitative validation through metabolic-flux analysis.

#### 7.3.4 Artificial nitrogen fixation: proof of concept and practical challenges

Engineering functional nitrogenase systems into non-legume crops faces three physiological barriers: oxygen sensitivity, metal-cluster assembly complexity, and high energy requirements (~16 ATP per N_2_ fixed). Jiang et al. (2022) achieved functional expression of NifB, a critical nitrogenase-maturation protein, in the chloroplasts and mitochondria of *Nicotiana benthamiana*, completing the initial assembly of the FeMo-cofactor module in these organelles. However, autonomous artificial nitrogen fixation would impose substantial energy demands under salt stress, when SnRK1-mediated energy-conservation responses are activated. Consequently, enhancing associative nitrogen fixation through engineered rhizosphere synthetic microbial communities (SynComs) may be a more practical alternative (Cheng et al., 2026).

Precision genome editing and synthetic biology can translate knowledge of signaling pathways and elite alleles into targeted genetic designs. **Table 3** compares these breeding strategies. Their application may support the development of crops adapted to salinized agroecosystems.

**Table 3:** Genetic resources and design strategies for breeding salt-tolerant and nitrogen-efficient crops.

Breeding strategy	Key gene/QTL	Target species	Phenotypic gain	Mechanism of action	Core challenges	Core references
QTL linkage mapping	Multiple NUE-associated QTLs	Maize, Barley, Rice	Moderate NUE improvement under combined salt and low-nitrogen conditions	Identifies genomic regions tightly linked to salt tolerance and NUE traits	Low mapping resolution; severe linkage drag	Agrama et al., 1999; Han et al., 2016
Genome-wide association study (GWAS)	*OsNRT1.1B*, *LHT5*^HapA^	Rice	10%–15% NUE enhancement under salt stress	Mines elite natural allelic variations from diverse germplasm resources	Population structure bias; poor detection efficiency for rare variants	Phan et al., 2023b; Wang et al., 2026
Wild germplasm introgression	*LHT5*^HapA^, halophyte-derived transporter alleles	Rice	Enhanced salt tolerance and novel NUE-related genetic variation	Introgression of stress-tolerant rare alleles from wild crop relatives	Hybrid sterility; widespread linkage drag from wild genomes	Wang et al., 2026
CRISPR-Cas9 gene knockout	*ARE1*, *OsRR22*	Rice	10%–20% higher grain yield under salt and low-nitrogen stress	Knocks out negative regulators to simultaneously improve NUE and salt adaptation	Off-target effects; unpredictable pleiotropic phenotypes	Wang et al., 2021c; Sheng et al., 2023
CRISPR-Cas9 HDR allele replacement	*OsNRT1.1B*	Rice	Improved nitrate uptake and salt adaptability under low-nitrogen conditions	Precisely replaces inferior japonica allele with high-efficiency indica allele	Extremely low HDR efficiency in cereal crops	Li et al., 2018
Multiplex genome editing	13 functional genes (SR86M)	Rice	Stable salt tolerance and compensated yield under 1.2% NaCl stress	Pyramids multiple stress-tolerant and high-efficiency agronomic traits	Antagonistic pathway interactions; potential off-target risks	Hao et al., 2025; Li et al., 2026
Cytosine base editing	*OsNRT1.1B* (Thr327)	Rice	Significantly improved NUE under salt stress via single amino acid modification	Modifies protein substrate affinity through precise single-site mutation	Limited editable genomic scope and base conversion types	Lu and Zhu, 2017
Genomic selection	Genome-wide molecular markers	Wheat, Maize	NUE trait prediction accuracy high	Captures minor-effect loci and models genotype-by-environment interactions	Limited training population diversity; high phenotyping cost	Crossa et al., 2016; Budhlakoti et al., 2022
Synthetic biology engineering	*SsNRT2.5*	*Arabidopsis*, Rice	Elevated nitrate accumulation and reduced Na^+^ toxicity under salinity	Enables stress-specific gene expression to avoid constitutive energy consumption	Unstable regulatory circuits under field conditions; strict biosafety constraints	Liu et al., 2026b; Cheng et al., 2026

## 8 Research bottlenecks and future perspectives

Despite progress in elucidating efficient nitrogen utilization under salt stress, the field faces three interconnected bottlenecks that constrain the translation of mechanistic knowledge into agronomic applications: systemic integration, dynamic regulation, and field translation. First, the systemic integration of salt-nitrogen crosstalk remains fragmented, as most studies focus on individual pathways rather than coordinated networks. Second, the spatiotemporal regulatory network, including transient PTMs and metabolic flux reprogramming, is insufficiently resolved, limiting the precision of trait manipulation. Third, a gap persists between laboratory findings and field performance, largely because genotype × environment × management (G×E×M) interactions. Addressing these bottlenecks requires integrating single‑cell omics, systems modeling, and precision agronomic management to establish a predictive framework for breeding.

### 8.1 Breaking the systemic integration bottleneck: single‑cell and multi‑omics technologies

Resolving the systemic physiological disruptions described in **Figure 2** requires the systematic deconvolution of the closed‑loop regulatory networks outlined in **Figure 1**. Single‑cell spatial omics and isotope‑tracing metabolic flux analysis (MFA) provide resolution for dissecting cell‑type‑specific responses and dynamic flux reprogramming.

Spatial transcriptomics (Stereo‑seq) has enabled high‑resolution mapping of cell types and marker genes in plant roots (Liu et al., 2025). Applying this technology to crop roots under salt stress could delineate the spatial expression patterns of *NRT* and *AMT* family transporters across the epidermis, cortex, and stele. Complementing this spatial information, single‑nucleus RNA sequencing (snRNA‑seq) revealed pronounced transcriptional remodeling in wheat root-hair cells under salinity, with HKT‑family sodium transporters specifically enriched in these cells. These findings indicate functional differentiation among cell types governing ion homeostasis and nutrient uptake (Du et al., 2026).

At the metabolic flux level, ^13^C‑and ^15^N‑isotope‑tracing MFA has enabled the simultaneous quantification of carbon and nitrogen fluxes in microbial systems and may be applied to investigate plant salt‑nitrogen crosstalk (Slater et al., 2023). This approach could resolve three quantitative uncertainties: the partitioning of nitrogen between primary amino-acid assimilation and osmoprotectant biosynthesis, such as proline production; the extent to which carbon-skeleton availability constrains GS–GOGAT cycle flux; and the contribution of the alternative GDH shunt to ammonium assimilation under stress. Transitioning from static metabolite profiling to dynamic flux analysis, combined with genome-scale metabolic modeling, should help address the systemic-integration bottleneck.

### 8.2 Bridging the translation gap: G×E×M modeling and multi-environment validation

Functional genes and elite alleles identified under controlled greenhouse conditions frequently exhibit attenuated or inconsistent phenotypic effects in heterogeneous field environments, largely because of poorly characterized G × E × M interactions. As discussed in Section 6.1, constructing standardized field phenotyping databases across multiple years, locations, salinity gradients, and nitrogen regimes, and integrating these datasets with multi-environment trial GWAS (MET-GWAS), is essential to evaluate the genetic stability and breeding utility of candidate loci (Crossa et al., 2016). Machine-learning algorithms should incorporate agronomic management variables, such as precision fertilizer regimes and irrigation dynamics, to construct comprehensive G × E × M prediction models. For genome-edited materials targeting hubs such as *OsNRT1.1B* and *ARE1*, rigorous multi‑salinity, multi-year field trials are indispensable to assess the environmental robustness and potential yield penalties of target traits before commercial deployment.

### 8.3 Construction of an agronomic-molecular-microbial management system

Genetic improvement alone cannot guarantee stable yield gains in highly heterogeneous saline farmlands. An integrated management system combining genetic improvement, agronomic management, and microbial enhancement may provide a practical route for translating these advances to saline agriculture.

At the genetic level, multiplex genome editing and synthetic biology can generate salt-tolerant, high-NUE germplasm. At the agronomic level, precision management, such as reducing nitrogen fertilizer application to one-quarter to one-half of the conventional rate, can improve nitrogen uptake and agronomic NUE, with greater benefits reported for salt-tolerant cultivars (Phan et al., 2023b). Additionally, targeted exogenous application of NO donors or melatonin may prime antioxidant defenses, thereby alleviating salt-induced oxidative damage and helping maintain ion homeostasis (Zhao et al., 2018). At the microbial level, the rhizosphere microbiome may help buffer environmental stress. For instance, the root endophyte *Bacillus siamensis* BT-9–1 restores rhizobial carbon and nitrogen metabolism under saline-alkali stress through metabolic cross-feeding, thereby enhancing biological nitrogen fixation (Ai et al., 2026). This supports the potential use of synthetic microbial communities (SynComs) as a complementary strategy to regulate salt–nitrogen interactions under field conditions.

Combining elite mechanism-driven cultivars with salt-tolerant plant growth-promoting rhizobacteria and precision fertilization may support the development of resilient production systems for salinized agroecosystems.

### 8.4 Beyond current paradigms: speculative frontiers and system‑level integration

The preceding sections described available approaches, including multiplex genome editing, salt-triggered synthetic genetic circuits, and rhizosphere microbial consortia, as well as their potential near-term applications in saline agriculture. This section focuses on long-term interdisciplinary directions that remain insufficiently validated experimentally. Rather than recapitulating the established vector systems, stress-inducible promoters, and single-microbe inoculation schemes discussed above, we outline three conceptual directions that extend beyond simply combining existing technologies. These directions require the integration of multi-omics profiling, synthetic biology, and plant microbiology and are likely to remain exploratory over the next decade.

#### 8.4.1 From gene stacking to network topology engineering

The multiplex CRISPR editing system described above typically applies an additive strategy in which several favorable genes are edited simultaneously, often without fully accounting for interactions among salt–nitrogen signaling pathways. This approach may result in growth–defense trade-offs and diminishing agronomic returns.

The long-term objective is to shift the engineering target from individual genes to the topology of the salt–nitrogen regulatory network. Three research prerequisites are needed: (1) single-cell spatial multi-omics mapping to resolve cell-specific dynamic interactions between Ca^2+^ signaling and carbon/nitrogen fluxes; (2) machine-learning predictive models to identify sensitive network nodes associated with coordinated stress responses; and (3) orthogonal synthetic regulatory cassettes to rewire endogenous signaling cascades rather than merely overexpressing individual transporters or transcription factors. The key distinction from the additive gene-stacking logic lies in shifting the engineering target from discrete gene modifications to network-level reconstruction rather than simply combining known elite alleles. This network-centered design has not yet been validated in staple crops and therefore remains a long-term research direction.

#### 8.4.2 From stress‑inducible binary switches to multi‑signal autonomous decision circuits

Salt-responsive promoters typically mediate on/off activation of nitrogen transport in response to salt signals but cannot integrate nitrogen concentration, carbon supply, and plant developmental signals to coordinate resource allocation.

The next generation of crop circuits may incorporate multi-input logic-gate systems (AND/OR/NOT) that integrate multiple inputs to regulate the relative allocation of resources to nitrogen assimilation and osmoprotectant synthesis under combined stress conditions, thereby potentially reducing continuous energy consumption associated with constitutive expression. At present, such modules have largely been limited to single-stress responses in model plants, and multi-signal integrated decision circuits have not yet been deployed in field crops exposed to combined saline stresses. This direction extends binary stress-induction tools by shifting from stimulus-responsive regulation towards context-dependent resource allocation.

#### 8.4.3 From crop‑centric breeding to holobiont co‑design systems

Current agronomic and microbial improvement strategies generally treat crops as the primary breeding target and rhizosphere microorganisms as external inputs, with limited consideration of bidirectional metabolic interactions between plant roots and associated microbial communities in saline, low-nitrogen soils.

The holobiont co-design paradigm treats plant–microbe symbiosis as an integrated breeding target. Two largely unexplored research directions are: coordinated editing of plant root-exudate genes and microbial nitrogen-fixation modules to establish bidirectional signaling systems; and construction of holobiont genomic prediction models to jointly evaluate the salt tolerance and nitrogen use efficiency of host–microbiome combinations. Unlike additive microbial supplementation schemes, holobiont design aims to promote coordinated plant–microbe adaptation. Its implementation will require high-throughput phenotyping platforms for plant–microbe symbioses, which are not yet available for routine agricultural applications.

The three research directions outlined above differ conceptually from the established technical systems and near-term optimization strategies discussed in the preceding sections. All three paradigms rely on the integration of single-cell omics, computational modeling, and synthetic microbial engineering and face substantial experimental and translational barriers that cannot be addressed by any single existing tool. These long-term directions may provide a conceptual basis for developing salt-tolerant, nitrogen-efficient crops adapted to heterogeneous saline farmland.

## 9 Conclusion

Crop NUE under salt stress is a multilayered regulatory process encompassing transporter dynamics, signal transduction, transcriptional regulation, and systemic carbon–nitrogen metabolic reprogramming. In this review, we have synthesized the molecular regulatory networks governing salt–nitrogen crosstalk and constructed an integrated regulatory framework. This framework integrates key regulatory layers, from plasma membrane perception to nuclear transcriptional feedback, linking physiological metabolism to genomic engineering. Within this framework, NRT/AMT transceptors, the ROS–Ca^2+^–MAPK signaling axis, transcription factor hubs (e.g., NLP and NAC), and the TOR–SnRK1 energy-sensing module collectively contribute to maintaining the balance between nitrogen assimilation and osmotic defense.

Recent technological advances, including single-cell multi-omics, spatial transcriptomics, and isotope-tracing metabolic flux analysis, have enabled investigation of salt–nitrogen responses at cellular resolution beyond tissue-level measurements. Concurrently, crop breeding has expanded beyond conventional QTL-based introgression to include CRISPR-mediated multiplex engineering and synthetic biology. The iterative design–build–test–learn (DBTL) cycle, coupled with machine-learning-driven G × E prediction models, may help address the genetic bottlenecks of traditional breeding, enabling the pyramiding of resilient traits at the genomic level.

Bridging the gap between molecular mechanisms and field application remains a major challenge. We advocate for a translational research pipeline that integrates systems biology, AI-assisted genome design, and precision agronomic management. Developing crop varieties capable of efficient nitrogen utilization in salinized soils is important for improving agricultural productivity while reducing fertilizer inputs and associated environmental costs. Ultimately, translating knowledge of salt–nitrogen regulatory networks into breeding frameworks may support sustainable agricultural productivity under climate change and increasing soil salinization.
